# Virtual Reality for Basic Life Support Training in High School Students: Thematic Analysis of Focus Group Interviews

**DOI:** 10.2196/53212

**Published:** 2024-04-16

**Authors:** Hyojin Jennifer Min, Caroline Andler, Rebecca Ortiz La Banca Barber, Todd P Chang, Cristian Abelairas-Gomez, Laquanda T Knowlin, Deborah R Liu, Nino Fijačko

**Affiliations:** 1 Children’s Hospital of Los Angeles Los Angeles, CA United States; 2 Faculty of Education Sciences Universidade de Santiago de Compostela Santiago de Compostela Spain; 3 Faculty of Health Sciences University of Maribor Maribor Slovenia

**Keywords:** virtual reality, mixed reality, technology, basic life support, cardiovascular pulmonary resuscitation, near-peer mentoring, education, high school students

## Abstract

**Background:**

High-quality and engaging cardiopulmonary resuscitation (CPR) training of both health care professionals and members of the public is necessary to provide timely and effective CPR to maximize survival and minimize injuries. Virtual reality (VR) is a novel method to enhance CPR engagement and training. However, a near-peer mentoring framework has not been applied in such training to date.

**Objective:**

The purpose of this pilot qualitative study was to understand the acceptability and feasibility of using VR technology to introduce basic life support (BLS) to high school students reinforced by near-peer coaching.

**Methods:**

Dyads of high school students underwent BLS training in CPR using a VR experience reinforced by the near-peer mentoring model. Focus group interviews were performed following the intervention. The interview sessions were recorded, transcribed verbatim, and subjected to thematic analysis. VR software data were analyzed after five cycles of chest compressions between the two participants.

**Results:**

The overwhelming responses from the three dyads of high school students indicated positive acceptance of learning CPR using VR. Analysis of emerging themes revealed three main categories of barriers and facilitators: (1) motivation to learn CPR, (2) CPR learning modality, and (3) coaching CPR content. These themes supported the theoretical framework of an “intention-focused” paradigm leading to acquiring the skills needed to perform CPR and ultimately increasing the chances of a bystander performing CPR.

**Conclusions:**

This study highlights the potential for training a unique population to increase bystander effects using novel VR technology coupled with a near-peer mentoring method. Further research is warranted to measure the outcome of the knowledge attained and the intention to perform CPR by high school students who participate in CPR education using VR and a near-peer mentoring method.

## Introduction

Sudden cardiac arrest (SCA) is an uncommon phenomenon in youth; however, according to the Centers for Disease Control and Prevention, approximately 2000 healthy people under 25 years of age in the United States die each year due to SCA [1]. Regardless of one’s age, the survival odds of SCA outside of a hospital setting are low, which is likely related to the low bystander cardiopulmonary resuscitation (CPR) rates [2,3]. Therefore, it is important to increase the general public’s awareness and knowledge levels of CPR to consequently increase the number of bystanders who may initiate CPR in an emergency situation [4]. Educating and engaging adolescents in life-saving maneuvers such as CPR is a crucial step in increasing the lifelong ability and motivation to take actions in an emergency [5,6].

High-quality and engaging CPR training of both health care professionals and members of the public is necessary to provide timely and effective CPR to maximize survival and minimize injuries [7]. Traditionally, CPR training has occurred in an in-person group setting utilizing CPR mannequins. Due to the COVID-19 pandemic, this traditional approach of CPR training shifted toward a focus on smaller group sizes to minimize hands-on simulation activities. Virtual reality (VR) is a novel method to enhance CPR engagement and training, which has shown rapid growth since 2019 [8,9]. The immersive VR training induces a greater sense of presence and agency when compared to traditional CPR training, and may be more effective in increasing the intention and initiative to perform CPR in real-world emergencies [10]. Such VR technology enhancements have been particularly well received by the “technology natives” of the younger generation [11]. A systematic review identified the current gap of this field as the lack of educational programs rated at 3-4 on the Kirkpatrick model, which is a method of evaluating the results of training and learning programs [12]. This highlights the importance of continued development and improvement in simulation education, particularly in the context of CPR training. While there is a growing interest in leveraging novel technologies for CPR training, few studies have assessed their potential [13].

Near-peer mentoring is a learner-centered model, where the pairing of mentors and mentees close in age and developmental stage allows for mentors to draw on personal experiences to connect with mentees. This facilitates connections and reflections integral to the experiential learning process [14]. However, the relationship between CPR training and use of VR technology based on a near-peer mentoring framework has not yet been investigated, particularly in the high school population.

To fill this gap, our primary objective was to assess the feasibility of VR technology as a novel learning modality for CPR training and to apply the near-peer mentoring model in this CPR training among adolescents. Toward this end, we evaluated the relationship between the following three domains: CPR, VR, and near-peer mentoring. Our secondary objective was to collect and document the lived experiences of adolescents upon experiencing VR to obtain basic skill sets associated with CPR.

## Methods

### Recruitment

High school students were recruited from participants of Camp Children’s Hospital Los Angeles (CHLA), which is an annual, week-long health care career exploration summer camp for high school students between 15 to 17 years of age in Los Angeles County. The VR-based CPR sessions were offered as a voluntary option. The study was conducted in the Las Madrinas Simulation Center at CHLA.

The target number of participants to recruit for this qualitative pilot study was not established a priori. According to Creswell [15], between 5 and 25 interviews for phenomenological studies were reported to be appropriate. In addition, Morse [16] specified recruiting at least 6 participants for phenomenological studies. Neither of these studies included the rationale for the indicated numbers.

### Ethical Considerations

The protocol and participant-facing materials underwent review by the CHLA institutional review board, and approval was obtained prior to any data collection (case number CHLA-22-00230). Informed consent and assent were obtained from the identified participants and their respective parents prior to the day of the study. Participants were not offered compensation. Consents and assents were electronically obtained via the Research Electronic Data Capture (REDCap) system where corresponding participant IDs were created. The identifiers were removed during the transcription process and the recordings were destroyed once transcription was completed.

### Data Collection

**Figure 1:**
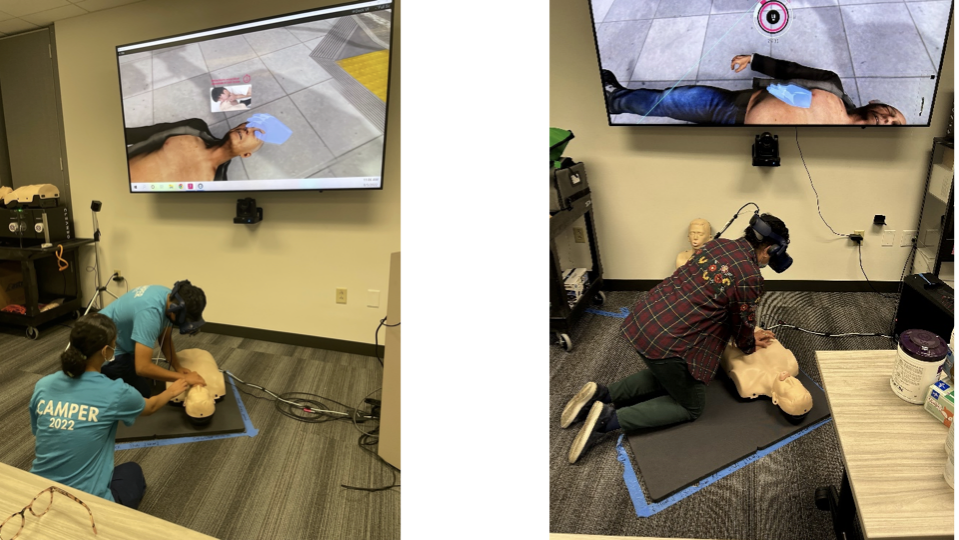
Dyads of campers underwent basic life support training using a virtual reality–based hybrid simulation platform.

Dyads of high school students underwent CPR training using a VR-based hybrid simulation platform (CBS, TetraSignum). Prior to the VR session, each dyad watched approximately 30 minutes of didactic content delivered by a virtual avatar instructor. Following the didactic portion, the students took turns and had an opportunity to perform hands-on CPR on a quality CPR (QCPR) mannequin. Next, the VR software data, which scored five cycles of chest compressions between the two participants, were analyzed. We used a Vive Pro (HTC) hardware system, which enabled simulcasting the VR user’s view to a screen for others to watch. The VR software superimposes a virtual avatar over the location of the mannequin to simulate a human in cardiac arrest. This QCPR technology uses wireless sensors embedded in the mannequin to measure the effectiveness of core CPR components [17]. The steps of the CPR consisted of (1) a check response, (2) a call for help, (3) a check for breathing, (4) five cycles of chest compressions and rescue breaths, and (5) using an automated external defibrillator. This experience was reinforced by the near-peer mentoring model as a pair (Figure 1). The sessions were immediately followed by approximately 45 minutes of focus group interviews led by the research team. The debrief interview sessions used open-ended questions addressing the domains of interest and the participants’ lived experiences. The interview sessions were recorded, transcribed verbatim, and analyzed.

### Phenomenology

Phenomenology is a type of a qualitative research method with roots in psychology and philosophy. Phenomenology is best applied to understand the lived experiences of individuals where the focus is exploring [18,19]. In our study, participants interested in a career in health care intentionally chose the VR CPR tract as part of their day. Accordingly, this study aimed to gain insight as to “how individuals make sense of the world to provide insightful accounts of their subjective experience” [18], and to gain understanding of the phenomenon of introducing the contents of basic life support (BLS) and CPR using VR and near-peer mentoring to high school students.

### Data Analysis

The interview sessions were recorded and transcribed verbatim. An interdisciplinary team of medical researchers used a thematic analysis approach as described by Braun and Clarke [20]. The first three authors (HJM, CA, and ROLBB) read the transcripts to understand the students’ perceptions and acceptability of using VR to learn CPR with a near-peer mentoring model. Next, the transcripts were coded systematically across the three team members and combined for reflexive thematic analysis [21]. The three research team members, including two research nurses (HJM and ROLBB) and a physician (CA), analyzed the focus group data using constant-comparison analysis. This approach allows for richer interpretations of meaning, particularly across multidisciplinary research members. Constant comparison also allowed for refining, defining, and naming themes. Once codes were created, they were grouped into barriers and facilitators, and then broader themes were identified by circling back to the near-peer mentoring model and the intersection between VR and CPR. Finally, the thematic auditors (DRL and TPC) reviewed the identified themes for any discrepancies.

## Results

### Recruitment

This pilot study launched over the summer of 2022. We recruited a total of three dyads of 6 high school students from a total of 31 students participating in the CHLA camp. The parents of the 6 participants provided consent and individual participants provided assent to be interviewed to share their lived experiences of learning CPR using VR and acting in the role of “coach” based on the near-peer mentoring model. The cohort comprised 2 boys and 4 girls with a mean age of 16.5 (range 15-17) years. Of note, this was the first on-site camp since the COVID-19 pandemic; therefore, there was a smaller total group of campers selected for that year, ultimately leading to a smaller sample size for this study.

### Data Saturation

Data saturation in a qualitative study is defined as the collection of qualitative data to a point of “sense of closure,” because there are no new insights obtained and the data yield redundant information [19,22]. Attempts were made to have the campers return to campus after the camp had been completed; however, since the students were back at school, we were unable to recruit additional participants.

### Themes

#### Main Themes Identified

**Figure 2:**
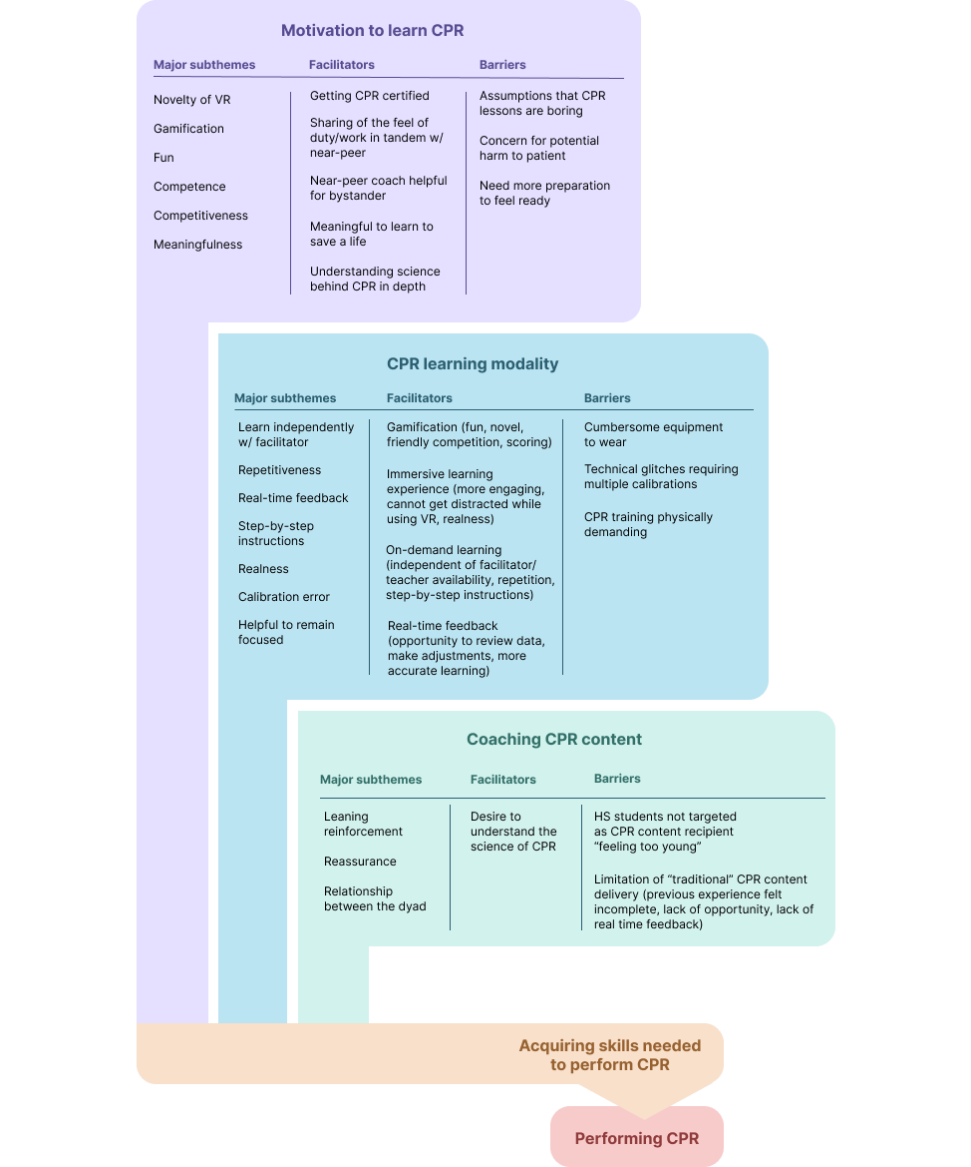
Summary of the thematic analysis. CPR: cardiopulmonary resuscitation; HS: high school; VR: virtual reality; w/: with.

During the qualitative analysis process, three themes were identified: (1) motivation to learn CPR, (2) CPR learning modality, and (3) coaching CPR content (Figure 2).

#### Motivation to Learn CPR

Motivation to learn CPR was defined by themes arising from the individuals’ expressed extrinsic and intrinsic motivation to learn. Participants reported the novelty of VR and the engaging space of the simulation center to be a motivating factor in learning CPR. Our method of learning was unique when compared to the traditional method of a CPR class and its preconceived notions because it created “a more engaging space for kids to be more interested in CPR.” Furthermore, the gamification of learning by VR was considered to be fun, and even peers who may not have an interest in health care would also be interested since VR would “automatically assumed to be fun because it’s a game.” The final scores provided by the software served as positive feedback of competence and elicited competitiveness as a source of encouragement in the participants’ ability to achieve success. Lastly, many of the participants reported that it was meaningful to be able to learn how to save a life.

#### CPR Learning Modality

The learning modality of CPR was defined by themes arising from the method, medium, or delivery of the CPR learning via VR and peer coaching. The participants reported that being able to learn independently without having a facilitator or teacher supervision would allow them to repeat the learning process at their own pace (eg, “you keep practicing, eventually it’ll become second nature”) and that when faced with an emergency, one would feel more prepared to assist. The real-time feedback from the avatar trainer and the in-person peer coach was considered to be helpful. In addition, the ability to address concerns such as “Are we going at the right pace?” “Are we going too slow?” “Is the pressure right?” on a real-time basis enabled immediately making changes and adjustments in real time as needed. The step-by-step instructions helped to reinforce the knowledge. One camper shared, “I like that it went step by step because it helped to absorb the information easier.” There was also feedback regarding the avatar often moving out of sync with little or no time for the participant to reflect. For instance, “it was like after you finish check response and then he just quickly moved on to the next one. Maybe just a little bit time to reflect…” Lastly, the realness of the VR simulation and tactile hands-on learning modality helped participants to be better prepared when compared to the traditional lecture-based or passive online modules: “I liked it was more accurate. I like that it put me in a real-life situation and, I felt like it made me more prepared.” The participants also shared calibration error to be a source of distraction in learning, which occurred when the mannequin’s sensor and the participant’s hands were not calibrated correctly. Lastly, use of VR enabled the participants to remain focused while learning, because “you weren’t looking around or getting distracted by anything,” since while the headset is on, the participant is engaged in the “VR world” and is therefore unable to become distracted.

#### Coaching CPR Content

Coaching CPR content was defined by themes arising from the dyads’ experiences of serving as a coach and being coached as a pair. Although the pair started by watching the 30-minute introduction training videos together, many participants shared that being able to coach after having the opportunity to go through the program first helped them to feel more confident in coaching. The comments offered as a coach served as additional learning reinforcement, as stated by one participant: “whatever I said to her stuck in my mind, so I was able to remember that for when I went.” The presence of the coach also provided a sense of reassurance because participants “felt reassured like I wasn’t being pressured to do everything and then forgetting anything.” Working in tandem in CPR training impacted the pair’s perception of the responsibility of a “scary situation” to be less daunting. An important factor to be mindful in assigning of the pair was assessment of the relationship between the dyad:

If I had just met someone, maybe I wouldn’t be as comfortable telling them what to do or what not. Probably just the relationship with people would be the biggest aspect of coaching.

#### Recommendations for Future Projects Provided by Participants

Helpful ideas for future iterations of the VR-based CPR trainings included adding options of different major metropolitan cities, background music, and personalization of avatars. Moreover, participants suggested expanding the trainings to include a pediatric population, ranging from infants to toddlers to school-aged children. Lastly, they suggested using a more seamless VR technology to impose less of a burden associated with the headset.

## Discussion

Our findings show that it will be valuable to leverage the currently available VR technology to promote CPR education for high school students. Moreover, purposeful inclusion of a near-peer mentoring approach can have a synergistic contribution to the training and result in a positive learning experience. The themes identified in our study build upon the existing theoretical framework proposed by Panchal et al [23], termed the “intention-focused model for bystander CPR performance,” which allows gaining an understanding of the determinants of bystanders’ decision-making process. The proximal domains preceding the intentions start with the bystander’s demographic characteristics, including gender, age, personality traits, and education level. These baseline demographic variables then lead to their “beliefs,” categorized by “attitudes,” “perceived norms,” and “self-efficacy,” in performing CPR. These beliefs then result in the “intention” to perform CPR, bolstered by the “skills needed to perform CPR” as a determinant of behavior, which ultimately leads to the action of performing CPR.

Therefore, the themes unveiled from our qualitative data analysis were consistent with the intention-focused model for the bystander CPR performance framework. Our research design of phenomenology and documentation of the “lived experiences” of the participants expanded upon the demographic characteristics and beliefs associated with CPR prior to this new learning experience. The main purpose of this VR CPR project was to equip the participants with the knowledge and skills needed to perform CPR. The three major themes unveiled from our analysis add to the preceding themes leading up to the “skills needed to perform CPR” in the intention-focused model, which were (1) motivation to learn CPR, (2) CPR learning modality, and (3) coaching CPR content. 

The motivation to learn CPR is a new domain that is distinct from the previously identified “attitudes,” “perceived norms,” and “self-efficacy” about performing CPR. Self-determination theory is a motivational theory of personality, development, and social process that examines how individuals are driven and depicts motivation on a continuum [24]. Notably, our participants were highly motivated individuals who are interested in future careers in health care. In accordance with the framework of Panchal et al [23], it will be important to introduce a moderating factor to motivate high school students to be intrinsically motivated where the motivation’s root stems from interest, enjoyment, and satisfaction.

The overwhelming positive feedback received by the participants in regard to learning CPR via VR is consistent with prior research showing a link between novelty and curiosity [11,25,26], where “when a novel stimulus affects an organism’s [brain] receptors, there will occur a driving stimulus producing response called curiosity” [26]. Our novel approach to the learning and delivery of CPR content may have steered the “attitudes” and “perceived norms” about performing CPR in a positive direction. A scientific statement by the American Heart Association noted that novel methodologies and digital platforms (ie, gamified learning, social media, and crowdsourcing) do not necessarily improve response and performance; however, novelty allows for the potential to reach a larger population with various types of learners [27]. Likewise, although this pilot study did not measure the changes in the “intention to perform CPR,” based on the feedback provided by participants, this approach has the potential to reach and create interest in high school students.

Since the VR CPR learning modality would allow for learners to repeat the learning sequences independently in their own time, as one participant stated, “if anything like this were to happen, you would be able to do it.” A similar study from 2016 that evaluated multiplayer virtual training in medicine among 12 Swedish medical students found that virtual training may result in “erroneous self-beliefs” affecting future clinical practices [11]. This study points to the importance of future studies to measure the “intention to perform CPR” and assessment of the efficacy of VR-based training compared to traditional training methods [13].

There was a similar study evaluating the influence of near-peer mentoring in CPR workshops on medical students’ knowledge and satisfaction [28]. Similar to the findings with our high school students, the previous study reported the benefits of this type of mentorship to be helpful in that the peers have similar levels of experience, and they are more familiar with the educational needs and better understand the learning process and potential areas for confusion [28].

The selection of inherently motivated high school students with career aspirations in health care may have posed a bias, thereby limiting the generalizability of the study, in addition to the small sample size of 6 participants. In addition, consistent with the design of this pilot study, it can be difficult to present generalizable findings of phenomenological research due to the highly individual records of lived experiences. Lastly, although we did not collect measures specifically evaluating changes in intentions to perform CPR in the future, previous studies have indicated that a bystander who had experienced CPR training was up to 6 times more likely to perform CPR when witnessing an out-of-hospital cardiac arrest [3,29,30]. Although outside of the scope and aims of this study, we did not collect any data to measure the quality of the CPR. Nevertheless, it is still meaningful that the students were trained on the sequences of BLS.

Our findings show that it is feasible to leverage a novel technology such as VR to enhance the CPR learning experience. Particularly for high school students, learning CPR using VR served as a source of motivation, which was fostered by the unique modality of learning in the presence of a near-peer coach. These benefits could contribute toward training a future generation who will be more confident to perform CPR as a bystander in an emergency situation.

## Abbreviations

BLS: basic life support
CHLA: Children’s Hospital of Los Angeles
CPR: cardiopulmonary resuscitation
QCPR: Quality CPR
REDCap: Research Electronic Data Capture
SCA: sudden cardiac arrest
VR: virtual reality

## Data Availability

Data for this study, which comprise the transcription files and coding from the first 3 authors, may be made available upon request to the corresponding author.
